# Research on Random Drift Model Identification and Error Compensation Method of MEMS Sensor Based on EEMD-GRNN

**DOI:** 10.3390/s22145225

**Published:** 2022-07-13

**Authors:** Yonglei Shi, Liqing Fang, Zhanpu Xue, Ziyuan Qi

**Affiliations:** 1 Department of Artillery Engineering, Army Engineering University of PLA, Shijiazhuang 050003, China; sylhbkd@hebust.edu.cn (Y.S.); qiziyuan2020@126.com (Z.Q.); 2 School of Mechanical Engineering, Hebei University of Science and Technology, Shijiazhuang 050018, China; xzp83@hebust.edu.cn

**Keywords:** MEMS sensor, random drift, error compensation, neural network

## Abstract

Random drift error is one of the important factors of MEMS (micro-electro-mechanical-system) sensor output error. Identifying and compensating sensor output error is an important means to improve sensor accuracy. In order to reduce the impact of white noise on neural network modeling, the ensemble empirical mode decomposition (EEMD) method was used to separate white noise from the original signal. The drift signal after noise removal is modeled by GRNN (general regression neural network). In order to achieve a better modeling effect, cross-validation and parameter optimization algorithms were designed to obtain the optimal GRNN model. The algorithm is used to model and compensate errors for the generated random drift signal. The results show that the mean value of original signal decreases from 0.1130 m/s^2^ to −1.2646 × 10^−^^7^ m/s^2^, while the variance decreases from 0.0133 m/s^2^ to 1.0975 × 10^−^^5^ m/s^2^. In addition, the displacement test was carried out by MEMS acceleration sensor. Experimental results show that the displacement measurement accuracy is improved from 95.64% to 98.00% by compensating the output error of MEMS sensor. By comparing the GA-BP (genetic algorithm-back propagation) neural network and the polynomial fitting method, the EEMD-GRNN method proposed in this paper can effectively identify and compensate for complex nonlinear drift signals.

## 1. Introduction

With the development of science and technology, information technology, and intelligence, sensors play an increasingly important role in social development. For example, in the field of navigation and flight control, UAVs use their own gyroscopes for attitude recognition [1,2]. In the field of vibration testing, acceleration signal is collected by acceleration sensor for vibration modal analysis. As early as 1961, Berg GV et al. studied the displacement characteristics of buildings through acceleration signals collected by sensors [3]. In the military field, it is also necessary to obtain acceleration signal by acceleration sensor in real time in the modification of one-dimensional trajectory model and dynamic parameter test of artillery [4]. In addition, sensors also play a huge role in medical, electronic consumption, and other aspects [5,6]. Traditional capacitive, piezoelectric, and other sensors usually cannot directly output digital signals and are relatively bulky. Due to the mechanical structure inside these sensors, they cannot withstand large overload shocks. The above shortcomings limit the use of this type of sensor in some occasions. With the development of semiconductor technology, MEMS sensors have also been greatly developed. Emerging MEMS sensors typically have the advantages of small size, high overload resistance, low cost, and low power consumption. Based on the above advantages, MEMS sensors are widely used in military, medical, electronic consumer, and other fields [7,8,9]. However, due to their physical structure and manufacturing accuracy, low-cost MEMS sensors often have output errors [10]. Therefore, the low cost of MEMS sensor output error limits its application range. For example, when displacement and Angle are obtained by using the acceleration or angular acceleration output by MEMS sensor, the existence of output error will be further amplified during quadratic integration [11,12,13]. Therefore, it is of great significance to compensate the output errors of MEMS sensors with low cost.

The random error of MEMS sensor is mainly caused by manufacturing accuracy, circuit interference, external environment, and other influence sources. The output error of MEMS sensors is usually not constant, so it is difficult to compensate quantitatively. The output error usually presents a complex nonlinear random drift characteristic. According to the characteristics of random errors of MEMS sensors, a variety of compensation methods have been studied, including time series analysis, polynomial fitting, and neural network compensation [14].

Time series analysis and Kalman filter are used to model and compensate the random drift signal output by sensors, and certain effects are achieved [15,16,17,18,19,20]. Time series analysis methods usually require the original random drift signal to have zero mean, stationarity, and normality. However, the original random drift signal is usually non-stationary, which requires first-order or multi-order difference processing on the original data to obtain a stationary signal. In addition, since the raw signals output by MEMS sensors are usually time-varying, it is difficult to describe them with a single time series model. It can be seen that the time series analysis method has relatively high requirements on the initial data, so it is difficult to realize real-time compensation.

Polynomial fitting is also a common method for modeling random drift errors of sensors [21,22,23]. If the random drift of the sensor is approximately linear or the drift trend is moderate, the error modeling by the polynomial fitting method can achieve a certain compensation effect. However, if the random drift of sensors presents complex nonlinear characteristics, the polynomial fitting method needs higher order to merge the drift errors and the fitting effect is usually not ideal.

As we know, neural network has powerful nonlinear function fitting ability. In the face of complex random drift signals, non-parametric identification based on neural network is a more effective method. BP neural network is one of the commonly used neural networks, and some related literatures also pointed out that the modeling effect of BP neural network is usually better than polynomial fitting method [24,25,26,27]. However, if the original drift signal is more complex, the function fitting ability of BP neural network will decrease. In addition, the BP neural network usually cannot reach the local minimum due to the improper selection of initial weights and thresholds in the training process [28]. In view of the shortcomings of BP neural network, some scholars proposed to use RBF (radial basis function) neural network for error modeling.

RBF network is a local neural network that converges to the global minimum. Many researchers pointed out that local neural networks have better modeling performance than global neural networks. In [29], a method of integrating RBF neural network and time series analysis for modeling was proposed. In fact, the complex and disordered white noise in the random drift signal of the sensor will affect the effect of neural network modeling. For example, a neural network polluted by noise is prone to “over-learning”. Considering this problem, some scholars have proposed the idea of modeling based on GRBFN (Grey-RBF) neural network [30,31]. The effect of white noise on the modeling efficiency of neural network is eliminated by cumulative generation operation. Although this method can eliminate the influence of noise to a certain extent by accumulative calculation, the complexity of calculation will increase with the increase of data volume and subsequent subtraction operation. In addition, the selection of recursive numbers in the modeling process also needs to be considered in depth.

In addition to the above methods, some researchers also improve the output accuracy by improving the physical structure and materials of sensors or designing compensation circuits. This method is highly specialized, difficult, and usually requires huge research funding [32,33,34,35]. In fact, the source of the output error of the sensor is multi-faceted and uncontrollable, so it is difficult to completely eliminate the output error by improving the hardware.

MEMS sensor errors include white noise, random drift errors, trend term errors, and constant value errors. After the white noise is separated from the original signal, the residual trend term, constant term, and random drift are modeled as a whole. The advantage of this method over time series modeling methods is that it avoids the complicated process of transforming the original signal into a stationary, random, zero-mean signal through multi-order difference.

Through the analysis of the above research status, it can be seen that first, the modeling effect of global neural network is better than that of local neural network. Second, the noise in the random drift signal of sensor will affect the modeling effect of neural network. Therefore, this paper considers the global neural network GRNN for error modeling after denoising the original signal output by the sensor.

The methods of separating noise from the original signal mainly include Fourier transform, wavelet multi-scale decomposition, moving average filter, and EMD (empirical mode decomposition). When the real signal is linear and has a different time or frequency scale from the noise, the Fourier filter can be used to separate the noise from the real signal. However, if the original signal is non-stationary, the filtering method will fail. Even if the real signal and noise have different fundamental frequencies, the harmonics of the fundamental frequency can still be mixed with the noise. This mixing of harmonic and noise will make Fourier filter an invalid noise separation method. The principle of noise reduction using wavelet multi-scale decomposition and EMD is to decompose the original signal into multiple components and remove the noise component. Although the wavelet multi-scale analysis method can decompose the signal and remove the noise signal, it needs to select a certain wavelet basis before the wavelet decomposition. The choice of wavelet basis function has great influence on the result of the whole wavelet analysis because the whole analysis process cannot be replaced after the wavelet basis function is determined. In addition, even though the wavelet base may be optimal globally, it may not be optimal locally [36,37,38,39]. Therefore, wavelet analysis lacks adaptability. The moving average filtering method is indeed a relatively easy and computationally inexpensive method. However, one obvious drawback is that the size of the sliding window has a significant impact on white noise removal. If the window length is larger, the locally averaged data will be more, and the smoothing effect will be larger. This is beneficial to suppress random errors of frequent random fluctuations, but it may also average and weaken the deterministic components of high frequency changes. Conversely, if the sliding window is small, it may be unfavorable to suppress low-frequency random errors. The above analysis shows that it is difficult for us to determine the size of the sliding window so as to eliminate noise precisely. Improper window size may eliminate useful signals other than white noise.

EMD is an adaptive signal decomposition method in the time domain and does not require the choice of basis functions, so EMD is very suitable for analyzing data from non-stationary and nonlinear processes [40]. The ensemble empirical mode decomposition method is developed on the basis of the EMD method. EEMD has many advantages over EMD in signal processing [41,42]. Therefore, this paper considers using EEMD to decompose the signal and then recombine the signal to achieve the purpose of noise reduction. The method of eliminating white noise based on EEMD has specific criteria. That is, after the white noise signal is decomposed by EEMD, multiple components will be obtained. The average period of each component is two times that of the previous one. In addition, in the process of decomposing the signal, the EEMD method does not need to subjectively set the decomposition parameters such as wavelet decomposition, etc. EEMD is a simple adaptive signal decomposition method, which has specific criteria compared to other methods for removing white noise. The specific principles and steps are described in detail in Section 3.3 below.

Since white noise will affect the modeling ability of the system, the white noise is first separated from the original signal by EEMD method according to certain principles. After denoising the original signal, GRNN can be used for error modeling. The optimal parameter model of GRNN can be obtained by cross-validation idea and parameter optimization algorithm. Therefore, the drift error modeling method based on EEMD-GRNN is finally proposed in this paper. This modeling method can improve the modeling effect of neural network, and has low requirement on the original random drift signal of sensor. The EEMD method for separating white noise and the characteristics of GRNN will be discussed in the following chapters.

This section will introduce the structural framework of this paper except the introduction part. The first part introduces the GRNN neural network algorithm and the method of GRNN parameter adjustment through optimization algorithm and cross validation. The second part introduces the EEMD algorithm and the principle of white noise separation. The third part introduces the application of EEMD-GRNN algorithm through a generated drift signal. The fourth part introduces the displacement measurement experiment based on acceleration integral. The influence of the sensor output error on the measurement results was analyzed and the measurement results were compensated. The last part summarizes the paper and presents the conclusion of the paper.

## 2. GRNN Structure and Parameter Optimization Algorithm

GRNN is an artificial neural network model based on nonlinear regression theory proposed by Professor Donald F. Specht of the United States in 1991. GRNN has strong nonlinear mapping ability and learning speed. GRNN can also achieve better prediction results when the sample data are small, and can also deal with unstable data. Although GRNN looks less accurate than radial basis, it actually has great advantages in classification and fitting, especially when the data accuracy is poor. GRNN is more suitable for solving curve fitting problems. In addition, the computational efficiency of GRNN is much faster than that of feed-forward neural network due to its simple structure [43]. Based on the above reasons, we choose GRNN to model and fit the sensor drift data.

### 2.1. The Structure of GRNN

**Figure 1:**
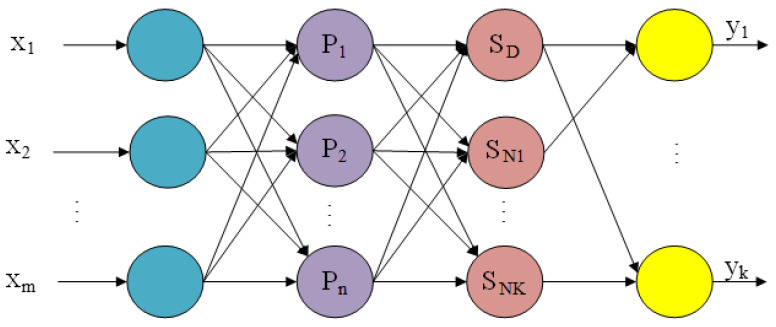
Schematic diagram of GRNN network structure model.

The GRNN network structure includes input layer, mode layer, summation layer, and output layer as shown in Figure 1. The input of the sample to the network is $X={\left[x_{1},x_{2}\ldots x_{m}\right]}^{T}$. The output of the sample from the network is $Y={\left[y_{1},y_{2}\ldots y_{k}\right]}^{T}$.

#### 2.1.1. Input Layer

The number of neurons in the input layer is equal to the dimension m of the input vector. Each neuron is a simple distribution unit whose role is to pass input variables directly to the mode layer.

#### 2.1.2. Mode Layer

The number of neurons in the mode layer is equal to the number of training samples n, and each neuron corresponds to a different training sample. The transfer function of the *i*-th neuron is:

(1) $$P_{i}=exp\left[\frac{-{\left(X-X_{i}\right)}^{T}\left(X-X_{i}\right)}{2\sigma^{2}}\right]\;i=1,\;2,\;...,\;n$$

In the above formula, X is the input sample of the neural network. $X_{i}$ is the training sample corresponding to the *i*-th neuron. *σ* is the smoothing factor of the neural network. The square of Euclid distance between X and $X_{i}$ is shown in formula (2).

(2) $$D^{2}{}_{i}={\left(X-X_{i}\right)}^{T}\left(X-X_{i}\right)$$

#### 2.1.3. Summation Layer

Two types of neurons are used for summation in the summation layer. One is the arithmetic sum of the outputs of all neurons in the mode layer. The weight value of neurons from the mode layer to the summation layer in the summation process is 1. The expression of its transfer function is:

(3) $$S_{D}=\overset{n}{\underset{i=1}{\sum }}P_{i}$$

The other is a weighted sum of neuron outputs in the mode layer. The transfer function of such neurons is:

(4) $$S_{Nj}=\overset{n}{\underset{i=1}{\sum }}y_{ij}P_{i},\;j=1,2,\ldots k$$

In the above formula, *y_ij_* is the connection weight of neuron *i* in the mode layer and neuron *j* in the summation layer.

#### 2.1.4. Output Layer

The number of neurons in the output layer is equal to the output dimension k of the training sample. The output of each neuron is the quotient of the two types of neurons in the summation layer as shown in the following formula:

(5) $$Y_{j}=\frac{S_{Nj}}{S_{D}},j=1,2,\ldots ,k$$

### 2.2. GRNN Parameter Optimization Algorithm

Although GRNN does not need training, the size of the smoothing factor σ has a great influence on the approximation accuracy of the network. The smaller the value of σ is, the stronger the network’s approximation capability to the sample is. However, the generalization ability of the network will become worse. The larger the value of *σ* is, the smoother the approximation process of the network to the sample data is, but the error also increases correspondingly. Therefore, constant adjustment is required to get the best *σ* value. In order to solve this problem, an optimization algorithm to find the best parameters of GRNN was designed.

The overall idea is to first conduct cross-validation to group the sample data, and then find the minimum prediction error value through the parameter cycle under each divided data set. The corresponding optimal parameter *σ* can be obtained through the minimum prediction error value finally obtained.

Cross-validation allows each sample to be used as a test set and training set. The neural network model can make full use of sample information through cross validation, which can ensure the robustness of neural network and prevent over fitting. There are many ways to perform cross validation, and k-fold cross validation was used in this article. The data set can be divided into K subsets by k-fold cross-validation, which enables each subset to be a test set and the rest to be a training set.

**Figure 2:**
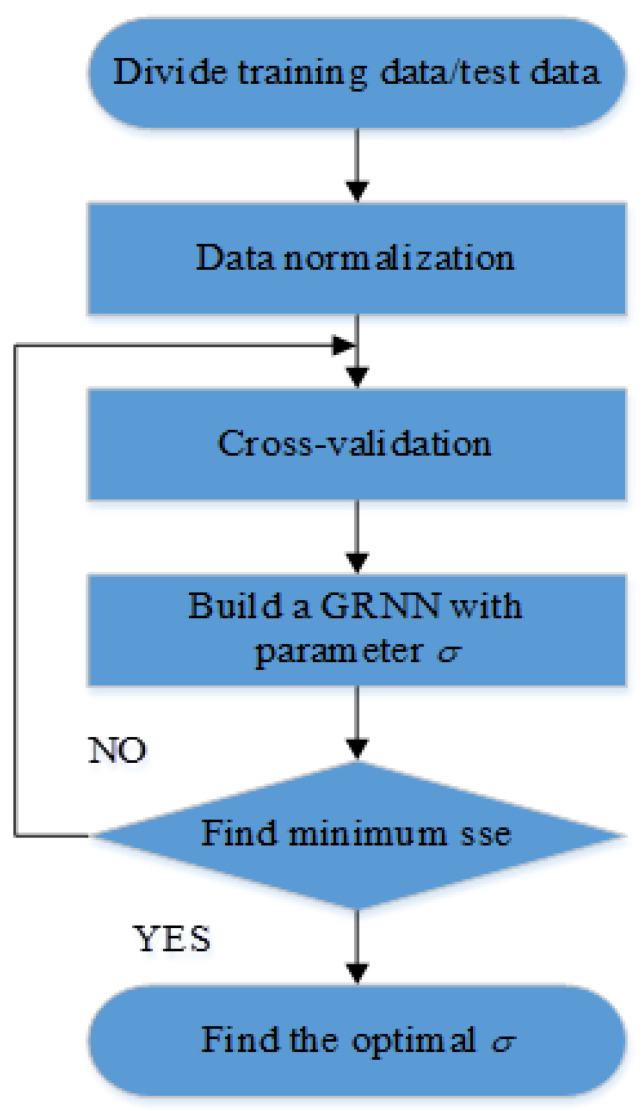
Program execution flow of GRNN parameter optimization algorithm.

First, we normalized the divided training data and test data. Then GRNN model with parameter *σ* can be established by MATLAB. The MSE (mean squared error) of the sample real value and GRNN predicted value under different parameters *σ* are continuously searched by cyclic instructions. The optimal *σ* value can be found by the minimum mean square error. The execution flow chart of parameter optimization algorithm is shown in Figure 2. The expression for MSE is shown in the following formula:

(6) $$\mathrm{MSE}=\frac{1}{M}\overset{M}{\underset{m=1}{\sum }}{\left(y_{m}-{\hat{y}}_{m}\right)}^{2}$$

In the above formula, *M* represents the number of input samples. $y_{m}$ represents sample output data, and ${\hat{y}}_{m}$ represents neural network prediction data.

## 3. White Noise Separation Method Based on EEMD

This part first analyzes the influence of noise on neural network modeling. Then the principle and function of EEMD in signal decomposition are introduced. Finally, the principle of separating white noise signal from original signal is given.

### 3.1. Influence of White Noise on Neural Network Modeling

**Figure 3:**
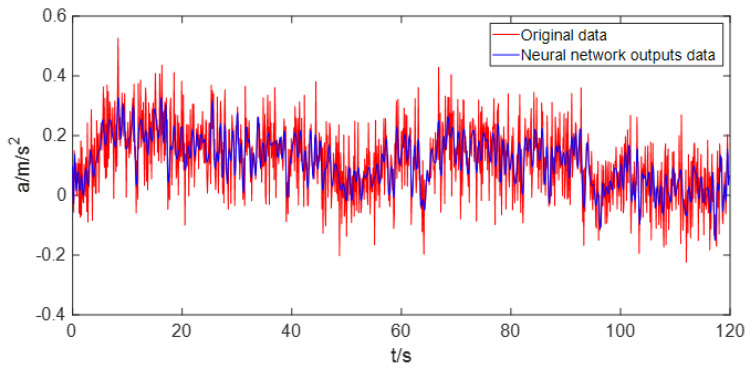
The output of the neural network is “polluted” by noise.

The output signal of MEMS sensor usually consists of white noise and random drift trend. The neural network is used to model a random drift signal, as shown in Figure 3. As can be seen from Figure 3, the neural network appears the phenomenon of “over-learning” due to the existence of white noise and unreasonable input and output data settings. The existence of noise will reduce the effect and efficiency of neural network modeling. In other words, the output of the neural network is polluted by the white noise of complex variation. Since noise has a certain impact on neural network modeling, the best way is to separate noise from the original signal before modeling the original signal.

### 3.2. EEMD Introduction and Principle of Signal Decomposition

EMD is a new adaptive time-frequency signal processing method suitable for the analysis and processing of nonlinear and non-stationary signals. EMD can perform signal adaptive decomposition according to the time scale characteristics of the data itself without the need to set the basis function in advance like wavelet decomposition. However, traditional EMD cannot decompose signals without enough extreme points, which further leads to the phenomenon of mode aliasing. In order to solve this problem, a signal decomposition method of EEMD has been proposed.

In recent years, EEMD is developed on the basis of EMD. In the process of signal decomposition, EEMD introduces the white noise with uniform spectral distribution into the signal to be analyzed, which can automatically distribute the signal to the appropriate reference scale. Because noise has the property of zero mean, it will cancel each other out after many times of averaging [44]. Therefore, the calculation result of the integrated mean can be directly regarded as the final result. EEMD can suppress mode aliasing effectively, so the decomposition effect is better than EMD. Based on the above reasons, this paper uses EEMD method to decompose the original signal in this paper.

The steps of the EEMD algorithm are as follows:

Step 1: Add normal distributed white noise to the original signal.

Step 2: Take the signal with white noise as a whole, and then perform EMD decomposition to obtain each IMF (Intrinsic Mode Function) component.

Step 3: Repeat Step 1 and Step 2, adding a new normal distributed white noise sequence each time.

Step 4: The result of IMF integration averaging is taken as the final result.

Ultimately EEMD can decompose arbitrarily complex signals into a finite number of intrinsic mode functions. 

### 3.3. Separation Method of White Noise

The white noise after EEMD decomposition is still a normally distributed signal, and each IMF is guaranteed to correspond to only one frequency value at each instant. That is, the IMF is a single-component signal. According to this feature, the different frequency components of the white noise signal can be decomposed into different IMF components.

It is generally believed that white noise is distributed in the first K IMF components, but there is no specific standard for the value of K. Therefore, certain criteria need to be established to separate white noise from the original signal. The statistical properties of white noise can be studied using the concept of averaging period [45]. The formula for the average period is:

(7) $$T=\frac{N}{n_{max}}$$

In the above formula, *N* is the number of sampling points and *n_max_* is the number of maximum points of the k-th order IMF. By calling the function “findpeaks” in MATLAB, the maximum number *n_max_* of the sequence can be obtained.

Since EEMD is a dichotomous filter [46], the average period of the IMF components obtained by decomposing signals with the same characteristics by EEMD can be followed regularly. That is, after white noise is decomposed by EEMD, the average cycle of each IMF is nearly twice that of the previous one. The original signal is decomposed by EEMD to obtain each IMF component. Based on the period of the first IMF component, if the subsequent IMF cycle is twice that of the previous one, the IMF component can be judged as a component of white noise.

## 4. Application of EEMD-GRNN Algorithm

**Figure 4:**
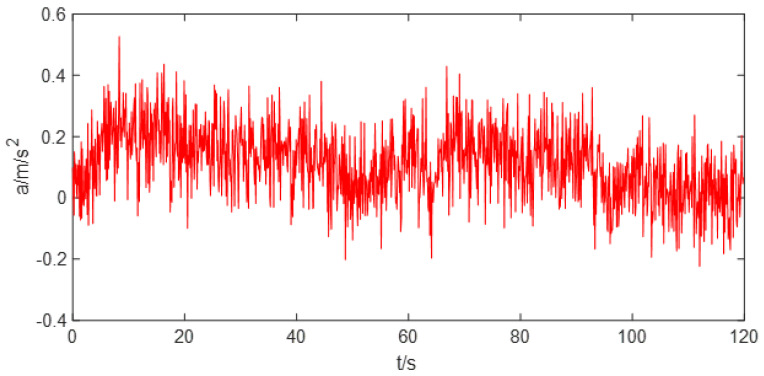
Raw signal with noise and random drift trends.

The application of EEMD-GRNN algorithm is introduced by processing a generated drift signal. First, the original signal is decomposed into multiple IMF components by EEMD signal decomposition method. The IMF component corresponding to white noise is found according to the criterion proposed in this paper. The drift signal without noise can be obtained by recombining the remaining signals. Then the optimal GRNN can be established by using cross validation and parameter optimization algorithm. The complex nonlinear drift signals can be modeled and fitted by using the optimal GRNN. A generated random drift signal is shown in Figure 4. Random drift signal contains white noise and random drift trend.

### White Noise Separation Method Based on EEMD

**Figure 5:**
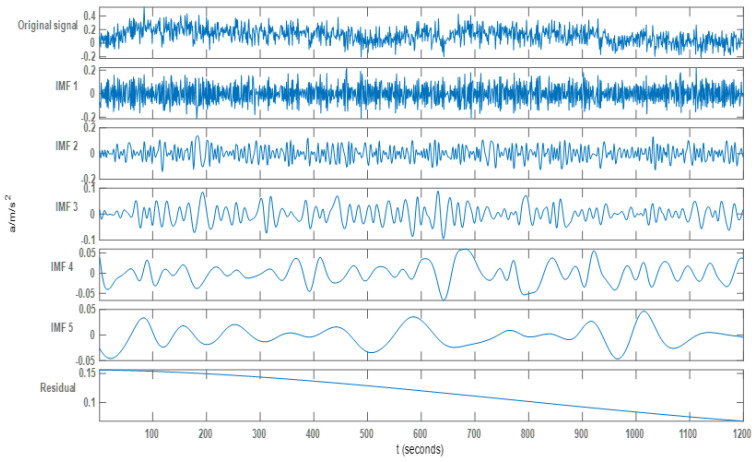
The first five component diagrams of the original signal after EEMD decomposition.

Each IMF component can be obtained after EEMD decomposition of the original drift signal. Each signal component after decomposition is shown in Figure 5. The original signal was decomposed by EEMD to obtain 10 IMF components. Due to space limitations, only the first 5 IMF components are shown in the figure.

After the original signal is decomposed by EEMD, the peak number and average period of each IMF component can be obtained by programming. The original signal was decomposed by EEMD to obtain 10 IMF components. The average period and peak number of each IMF component are shown in Table 1. In addition, the multiple relationship between the period of each IMF component and the period of the IMF1 component is also listed in the table.

**Figure 6:**
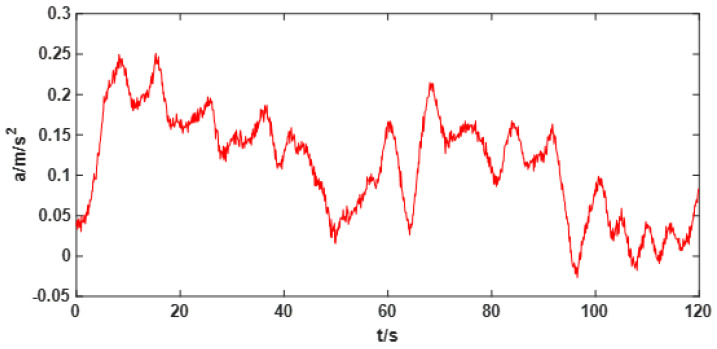
Drift trend signal after removing noise from original signal.

From Table 1, it can be clearly seen that from IMF1 to IMF4, the average period of each component is almost twice that of the previous one. According to the theory described above, it can be concluded that the first four IMF components are obtained by EEMD decomposition of white noise. Therefore, the drift trend without noise can be obtained by subtracting the first four IMF components from the original signal, as shown in Figure 6. It can be seen from Figure 6 that the drift signal after denoising has strong regularity.

## 5. Recognition Method of Denoising Signal

This part introduces the modeling method and process based on GA-BP and GRNN in detail, which is the application of some relevant theories and algorithms proposed above. In addition, the modeling effects of the two methods are compared in this section.

### 5.1. Moeling Method Based on GA-BP

**Figure 7:**
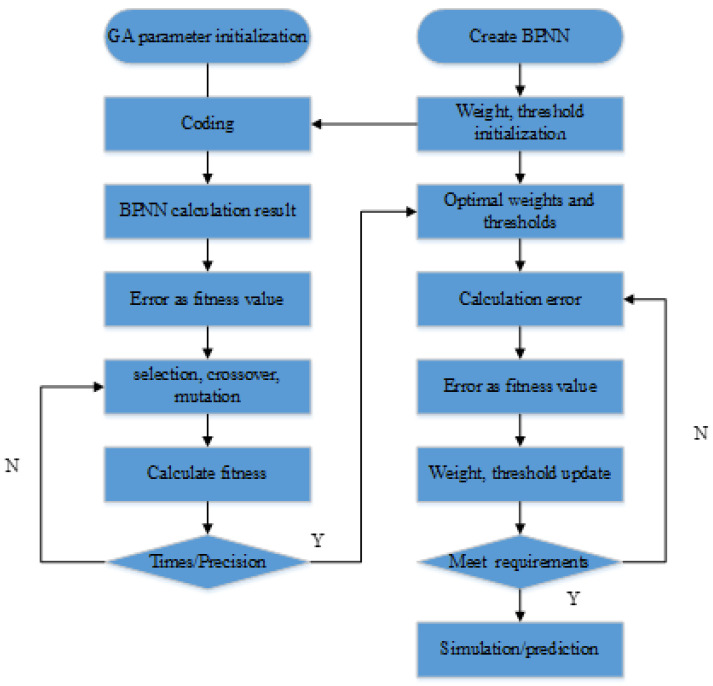
Program execution flow of the BP neural network optimized by genetic algorithm.

BP neural network is also one of the neural networks often used for regression and prediction. However, due to the random selection of initial weights and thresholds in the training process, it is easy to fall into the problem of local optimal solution. In order to improve the prediction accuracy, genetic algorithm is usually used to optimize the initial weights and thresholds [47]. First, a BP neural network is constructed and its parameters are initialized by genetic algorithm. Genetic algorithm can generate the initial weights and thresholds of BP neural network according to certain rules. The initial population was coded and the prediction error of BP neural network was used as fitness function. The optimal weights and thresholds of BP neural network can be found after the genetic algorithm stops according to the specified criteria. Taking the best weights and thresholds as the initial values of BP neural network parameters for training can achieve the best prediction effect. The whole GA-BP algorithm flow is shown in Figure 7.

**Figure 8:**
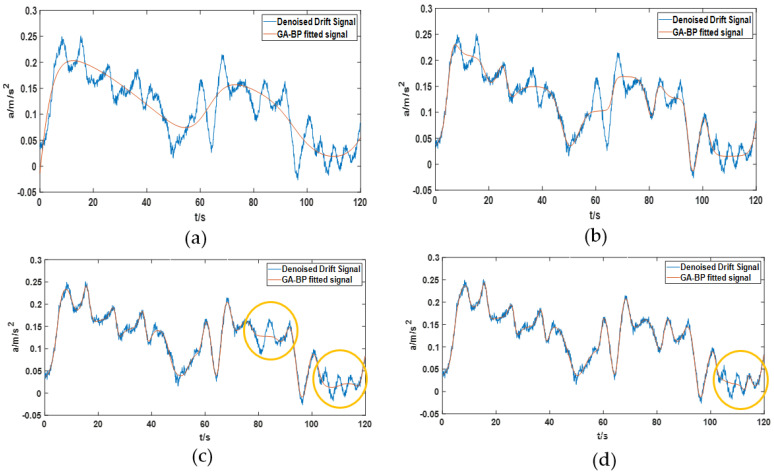
Modeling effect of GA-BP neural network on denoising signals. (**a**) A three-layer neural network with five neurons in the middle layer is used to model the denoised signal; (**b**) a three-layer neural network with 15 neurons in the middle layer is used to model the denoised signal; (**c**) a four-layer neural network with 25 and 10 neurons in the middle layer is used to model the denoised signal; (**d**) a four-layer neural network with 25 neurons in the middle layer is used to model the denoised signal.

GA-BP is used to fit the complex nonlinear drift signal after denoising, as shown in Figure 8. Figure 8 shows the modeling effect of the GA-BP neural network as the number of neurons and layers increase. Although the modeling effect of the GA-BP neural network becomes better with the increase of the number of neurons and layers, there is still a local modeling error as indicated by the yellow circle in the figure. It is worth noting that as the number of neurons and layers increases, the processor needs to pay more running time. In addition, multinomial fitting was also used for fitting. It is found that although the fitting polynomial order is very high, the fitting effect is still very unsatisfactory. Due to space constraints, polynomial fitting renderings are not listed here.

### 5.2. Modeling Method Based on GRNN

**Figure 9:**
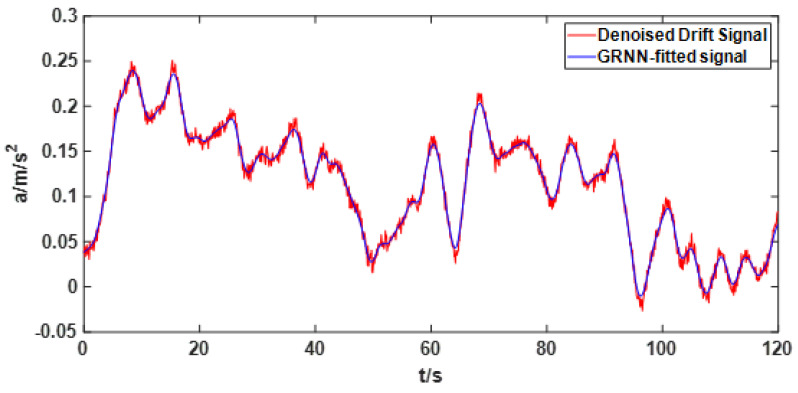
The fitting effect of GRNN on the drift signal after denoising.

First, the best GRNN model is obtained through cross-validation and parameter optimization algorithm. The original signal after noise removal is then fitted by this optimal GRNN model. The signal fitted by the best GRNN model is compared with the signal with noise removed from the original signal as shown in Figure 9. From Figure 9, it can be found that the signal fitted by GRNN better reflects the drift trend of the signal after noise reduction.

**Figure 10:**
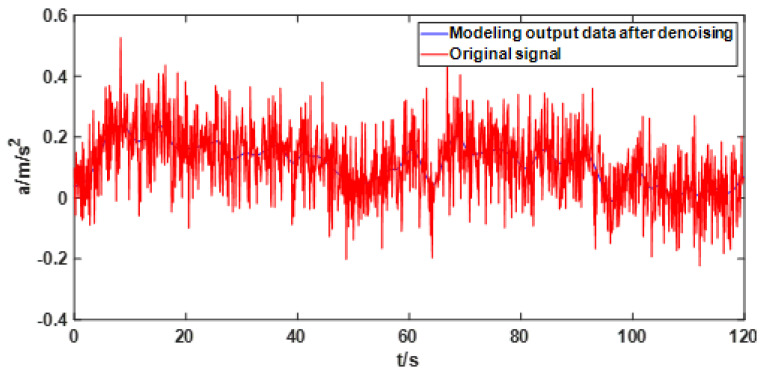
Comparison of original signal and GRNN modeling output signal.

In order to reflect the effect of GRNN modeling after noise reduction, the original data and the modeling output data of GRNN are compared, as shown in Figure 10. Compared with Figure 3, it is obvious that the output data after GRNN modeling is no longer affected by noise.

In order to have a more intuitive understanding of the modeling effect of GA-BP neural network and GRNN, the mean and standard deviation of the compensated signals and the running time of the processor in the operation process were compared, as shown in Table 2.

Through calculation, it can be obtained that the mean of the original signal is changed from 0.1130 m/s^2^ to −1.2646 × 10^−7^ m/s^2^, and the variance is reduced from 0.0133 m/s^2^ to 1.0975 × 10^−5^ m/s^2^.

## 6. Acceleration-Based Displacement Measurement Experiment

In order to check the effect of sensor error compensation before and after, a displacement test based on acceleration integral was carried out. This section first introduces the hardware system of acceleration data acquisition. Second, the displacement algorithm based on acceleration integral and the influence of sensor output error on the integral are introduced. Finally, the measurement accuracy of displacement after compensating sensor output error is given.

### 6.1. Design of Acceleration Acquisition System

**Figure 11:**
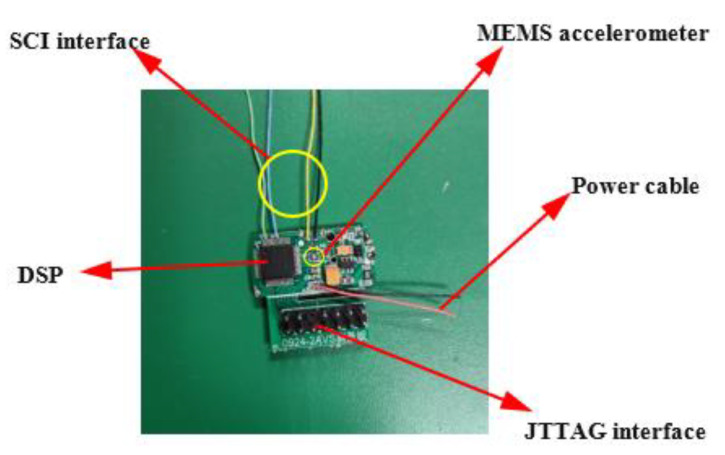
Physical hardware diagram of acquisition system and its composition.

The system consists of DSP microprocessor, MEMS acceleration sensor, power supply module and peripheral circuit. As the acquisition module of the system, the MEMS acceleration sensor is mainly used to sense the acceleration of the object in real time and transmit the acceleration data to the DSP through the IIC interface. In order to reduce system error, two sensors are integrated on the circuit board to collect data at the same time. A pair of accelerometers are located symmetrically on either side of the board. As the control module of the system, DSP microprocessor is mainly used for data processing and data storage. When the acceleration data is collected by the system, it can be further transmitted to the upper computer through SCI (serial communication interface) for data analysis. The hardware structure of the whole measurement system is shown in Figure 11.

**Figure 12:**
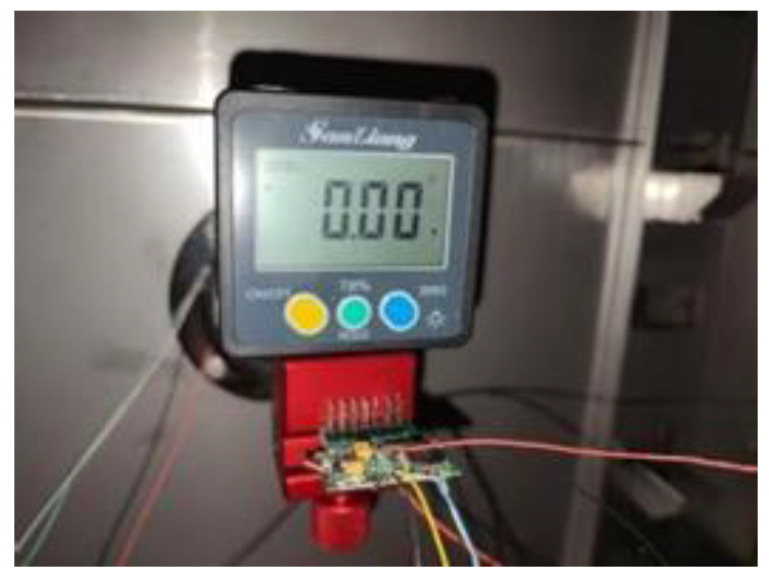
Accelerometer zero random drift test process.

The sensor can be adjusted to horizontal position by high precision dial and level for random drift test. In order to improve the stability of data acquisition process, the measuring board is positioned and clamped by a special fixture. The test device and data acquisition process of zero random drift test are shown in Figure 12. In order to reduce the error in the measurement process, the sampling system sampled at two horizontal positions of 0 degrees and 180 degrees respectively and took the average value as the drift error of the sensor.

### 6.2. Displacement Measurement Method Based on Acceleration Integral

Acceleration-based displacement measurement technology has a long history and has been applied in engineering practice [48]. The accelerometer based on MEMS technology has the advantages of small size, light weight, and strong impact resistance. Therefore, based on the need of measuring displacement in a research project, we adopt MEMS acceleration sensor to collect acceleration. DSP samples acceleration at a sampling frequency of 100 HZ, so the acceleration data collected are discrete. So, the displacement of the object can be calculated according to the discrete acceleration value.

According to the integral relationship between acceleration and velocity, the following equation can be obtained [49]:

(8) $$v(t)=\int_{t_{0}}^{t}a(t)dt+v(t_{0})$$

If the acceleration is a discrete value, the velocity value of any point can be obtained as follows:

(9) $$v\left[n\right]=\overset{n}{\underset{k=1}{\sum }}\frac{a\left[k\right]+a\left[k-1\right]}{2}\Delta t+v\left[0\right]$$

In the above formula, v[n] is the velocity of the object at any point, and a[k] is the discrete acceleration value at any point. Δt represents the time interval between two adjacent sampling points, and a[0] represents the initial acceleration of the object.

Similarly, according to the integral relationship between velocity and displacement, the following formula can be obtained:

(10) $$s\left(t\right)=\int_{t_{0}}^{t}v\left(t\right)dt+s\left(t_{0}\right)$$

When the discrete velocity value is obtained, the discrete displacement value can be further obtained as shown in the formula below:

(11) $$s\left[n\right]=\overset{n}{\underset{k=1}{\sum }}\frac{v\left[k\right]+v\left[k-1\right]}{2}\Delta t+s\left[0\right]$$

The velocity and displacement of a moving object at any time can be calculated by cyclic iteration algorithm.

### 6.3. Measurement Error Analysis

Although the displacement measurement method based on acceleration has been applied in many fields, the accuracy of measurement is severely restricted by the existence of sensor output errors. Therefore, it is necessary to explore the influence of sensor output error on displacement measurement. This section analyzes the influence of sensor output error on displacement based on displacement calculation formula. Assuming the sensor has a fixed output error ξ, the acceleration at any point becomes a[n]+ξ. The velocity with error can be obtained by substituting the acceleration with error into the discrete velocity calculation formula.

(12) $$v{\left[n\right]}^{{}^{\prime }}=\overset{n}{\underset{k=1}{\sum }}\frac{a\left[k\right]+a\left[k-1\right]+2\xi }{2}\Delta t+v\left[0]=v\left[n\right]+v[0\right]+\frac{1}{2}n\xi \Delta t$$

It can be found from the above formula that if there is an error ξ in the sensor output, the velocity error through one integration is nξΔt/2. Furthermore, the velocity expression with error is substituted into the discrete displacement formula to obtain the displacement expression with error.

(13) $$s{\left[n\right]}^{{}^{\prime }}=s\left[n\right]+s\left[0\right]+\frac{1}{2}n^{2}\Delta t^{2}\xi$$

The displacement error of acceleration error after quadratic integration is $n^{2}\Delta t^{2}\xi /2$. It can be concluded that when the sensor has output error, the error will participate in the integral calculation and be gradually amplified in the process. Therefore, it is necessary to compensate the output error.

### 6.4. Experiment

**Figure 13:**
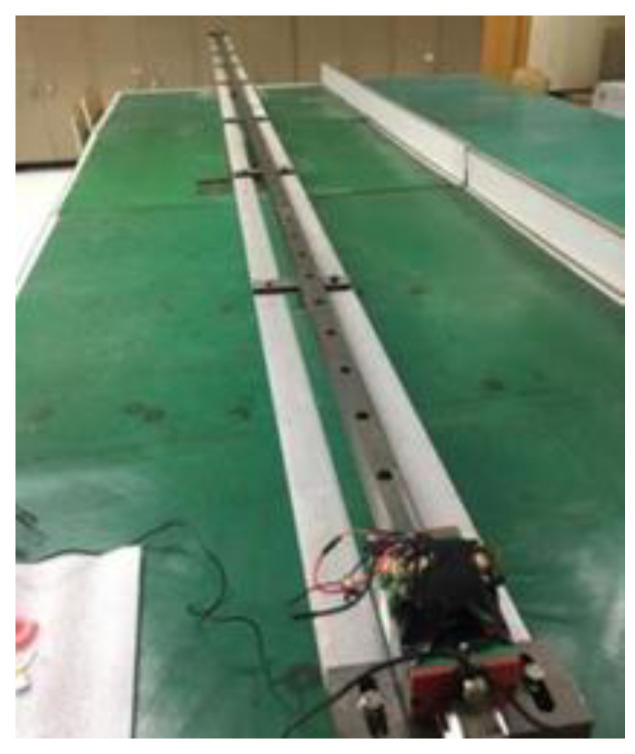
Device and process for acceleration acquisition by slider motion.

First the data acquisition system is placed on the horizontal slider as shown in Figure 13. A laser level is used to adjust the slide to a horizontal position. A small lithium battery is used to power the acceleration acquisition system during the test. When the slider starts to move with a certain acceleration from the rest, the acceleration sensor will collect the acceleration of the slider at a certain frequency in real time.

**Figure 14:**
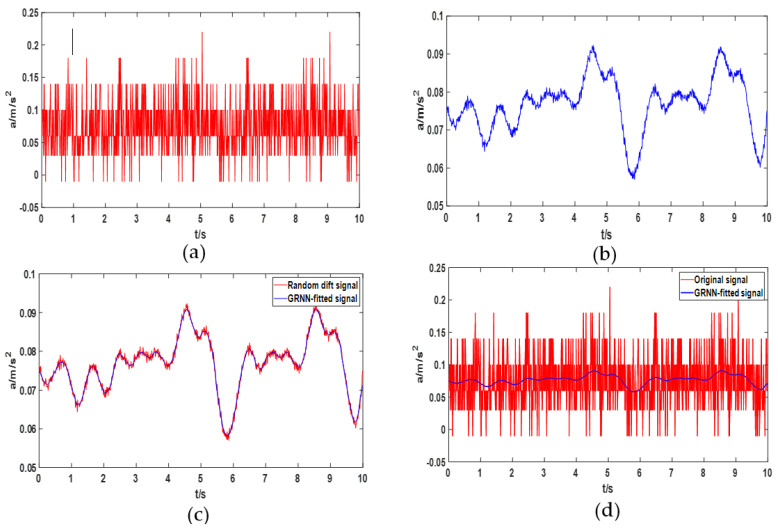
The original signal and its noise reduction results and the GRNN modeling process. (**a**) Original output signal of MEMS sensor; (**b**) random drift signal after denoising the original signal; (**c**) modeling random drift signals with GRNN; (**d**) comparison of original signal and GRNN-fitted signal.

After the test, the acceleration data during the slider movement can be obtained through the SCI of the DSP. The motion displacement of the slider can be obtained by the algorithm based on the acceleration integral given above. In addition, we present the original signal output by the sensor, the denoised signal and the comparison between them in Figure 14. At the same time, through MATLAB calculation we can get the optimal smoothing factor of GRNN is 0.01.

A total of five tests were carried out, and the actual movement displacement of the slider was set to 5 m during the test. Table 3 shows two displacement calculation results, one of which is calculated by the acceleration directly output by the sensor, and the other is calculated by the error compensation of the acceleration.

The test results show that the measurement accuracy of displacement is improved after compensating the output error of acceleration. Due to errors in the manufacturing process, the sensor sensitive axis and PCB board are not absolutely parallel, which may also cause errors in the displacement measurement. In addition, due to the sampling frequency of acceleration and other uncontrollable factors, the displacement measurement results still have certain errors. Due to the limited space of the experimental site, the displacement test distance is short, which leads to the compensation effect of the sensor output error which is not obvious. However, according to the expression of displacement error deduced above, we have reason to believe that the compensation effect will be more obvious with the longer working time of the sensor. In addition, we have done experiments with other sensors and found that the error output characteristics of different sensors are different.

## 7. Discussion and Conclusions

In this paper, the compensation method of random drift error of MEMS acceleration sensor was studied. The idea of neural network recognition and modeling after denoising the original signal was proposed in this paper. The measurement noise in drift signal is separated effectively by EEMD method. In addition, the optimal prediction model of GRNN was obtained by cross validation and parameter optimization algorithm. Compared with GA-BP neural network and polynomial fitting method, GRNN can effectively identify complex nonlinear random drift signals. Finally, the effectiveness of the compensation algorithm is verified by the displacement measurement experiment based on acceleration integration. Based on the research content of this paper, the following information and conclusions can be obtained:

1. Due to the unreasonable setting of input and output samples of neural network, the existence of noise will affect the modeling effect of neural network.
2. According to the characteristic of EEMD with binary filter, white noise can be separated from the original signal.
3. Neural network has better modeling effect for denoised signals.
4. The best GRNN model based on cross validation and parameter optimization algorithm has better fitting effect on complex nonlinear drift signals than BP neural network.
5. If the output error of the acceleration sensor participates in the integration process, it will be gradually amplified in the integration process.
6. The error compensation algorithm based on EEMD-GRNN can compensate the sensor output error to a certain extent. The low requirement for the raw output data of the sensor is also an advantage of this algorithm compared with other compensation algorithms.
7. Different sensors have different drift characteristics. The drift process of some sensors is more intense, while the drift process of other sensors is more gentle.

This paper only studies the compensation method of random drift error of sensor at normal temperature. The random errors of MEMS sensors may be affected by the temperature in extreme environments in addition to their own fabrication materials and physical structures. Therefore, the next step is to use the temperature control box to conduct experiments to study the error characteristics of the sensor at different working temperatures. If the sensor is significantly affected by temperature, it may be a good idea to incorporate random drift errors into temperature errors to compensate. In addition, other factors that cause sensor output errors still need to be further explored. The characteristics of the error signal output by different types of sensors may be different, so choosing the appropriate signal processing method can achieve the best compensation effect.

## Figures and Tables

**Table 1:** The relationship between the average period of each IMF and the multiple of IMF1.

IMF Component	Average Period	Number of Peaks	Multiple
IMF1	2.69	446	1.00
IMF2	5.50	218	2.04
IMF3	10.81	111	4.01
IMF4	21.81	55	8.10
IMF5	46.15	26	17.10
IMF6	100.00	12	37.17
IMF7	240.00	5	89.22
IMF8	600.00	2	223.05
IMF9	1200.00	1	446.09
IMF10	1200.00	1	446.09

**Table 2:** Comparison of compensation effects between BPNN (back propagation neural networks) and GRNN.

Method	Mean (m/s^2^)	Variance (m/s^2^)	Runtime (s)
Three-layer BPNN with 5 neurons	−6.3801 × 10^−4^	7.2346 × 10^−4^	0.3689
Three-layer BPNN with 15 neurons	−2.7316 × 10^−4^	2.4139 × 10^−4^	0.5988
Four-layer BPNN with 25 and 10 neurons	2.2853 × 10^−4^	7.2478 × 10^−4^	1.1990
Four-layer BPNN with 25 and 25neurons	1.1045 × 10^−4^	6.9435 × 10^−4^	3.7996
GRNN	−1.2646 × 10^−4^	1.0975 × 10^−4^	0.2567

**Table 3:** Calculation results of displacement before and after acceleration compensation.

Actual Displacement	Before Compensation	Measurement Accuracy	After Compensation	Measurement Accuracy
5 m	5.23 m	95.4%	5.10 m	98%
5 m	5.20 m	96%	5.09 m	98.2%
5 m	5.18 m	96.4%	5.11 m	97.8%
5 m	5.23 m	95.4%	5.08 m	98.4%
5 m	5.25 m	95%	5.12 m	97.6%

## Data Availability

Not applicable.
